# Advancing Equitable Ambulatory Telehealth Through Dashboard Development

**DOI:** 10.1089/tmr.2024.0033

**Published:** 2024-07-29

**Authors:** Leah R. Meisel, James P. Marcin, Zhong Wu, Kathryn M. Lopez, Mark Avdalovic, Jennifer L. Rosenthal

**Affiliations:** ^1^ Center for Health and Technology, University of California Davis, Sacramento, CA, USA.; ^2^ Department of Pediatrics, University of California Davis, Sacramento, CA, USA.; ^3^ Data Center of Excellence, University of California Davis, Sacramento, CA, USA.; ^4^ Division of Pulmonary, Critical Care and Sleep Medicine, University of California Davis, Sacramento, CA, USA.

**Keywords:** dashboard systems, digital health, health equity, telemedicine, quality improvement

## Abstract

Telehealth has the potential to improve access to health care by mitigating barriers related to geography, time, and finances. However, the increased adoption of ambulatory telehealth has inadvertently widened access gaps for socially disadvantaged and marginalized populations. Quality improvement approaches are a valuable strategy to address health care access inequities and disparities, involving data-driven implementation, assessment, and adaptation of tests of change over time. Because these iterative changes and interventions are data-driven, a critical element of quality improvement requires ongoing data collection and monitoring. This perspective describes the development and validation processes of a telehealth equity dashboard. This dashboard is currently available for use by our health system leaders, providers, and clinic staff. The overall objective of this dashboard is to identify and track inequities and to improve equitable ambulatory telehealth access across diverse patient groups. Lessons learned from creating this dashboard can inform other health care systems of how to develop and validate telehealth data feedback systems to promote quality improvement efforts to advance telehealth equity and accessibility.

## Introduction

Video visits are ambulatory telehealth encounters that leverage real-time, two-way audio and video communication between patients and health care providers.^1^ Video visits can enhance access to health care by mitigating barriers such as geography, time, and cost.^2^ Many individuals favor telehealth for its convenience, reducing clinic visits, and offering flexibility for those with mobility, scheduling, and caregiver challenges.^3,4^ Unfortunately, research demonstrates that the increased use of ambulatory telehealth following the COVID-19 pandemic has exacerbated telehealth access inequities among populations with advanced age, low income, limited English proficiency, and low digital literacy.^5–11^

Quality improvement (QI) methods are well-suited to address telehealth access inequities because the contributors to these inequities are primarily at the sociocultural and health care system levels.^12,13^ QI utilizes data-driven approaches to implement, assess, and adapt tests of change, necessitating continuous real-time data collection and monitoring. This perspective outlines the development and validation processes of a telehealth equity dashboard. This dashboard is currently available for use by our health system leaders, providers, and clinic staff. The objective of this dashboard is to organize data to support improvement efforts that enhance equitable access to video visits for diverse patient populations. The lessons learned can inform other health care systems in developing and validating telehealth data feedback systems to promote QI efforts to advance telehealth equity and accessibility.

## Designing a Dashboard for QI to Improve Equitable Telehealth Access

### QI requires local buy-in

Our team conducted a mixed methods evaluation of ambulatory telehealth encounters, demonstrating lower completion rates among groups preferring languages other than English and those with public insurance.^14^ The study’s qualitative phase identified strong provider buy-in to address these access inequities; providers desired systematic improvement initiatives to expand telehealth access. A key recommendation was to prioritize real-time data transparency and develop a user-friendly, widely accessible dashboard for telehealth to drive QI efforts.

### QI dashboard design

We formed a multi-disciplinary team to codesign an ambulatory telehealth equity dashboard. The team included members with expertise including QI, telehealth, digital equity, data curation, data validation, and project management. The dashboard includes three figures that each pertain to standard measures for assessing QI initiatives:

- Outcome measure: Successfully completed telehealth visits as a percent of all completed ambulatory visits
- Process measure: Scheduled telehealth visits as a percent of all scheduled ambulatory visits
- Balance measure: Failed telehealth visits as a percent of scheduled telehealth visits

**FIG. 1.**
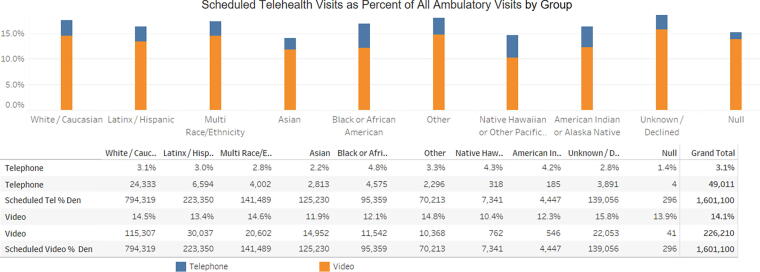
A graph of the process measure (“Scheduled Telehealth Visits as Percent of All Ambulatory Visits by Month”) stratified by patient race/ethnicity. The data are drawn from a random combination of clinics.

**FIG. 2.**
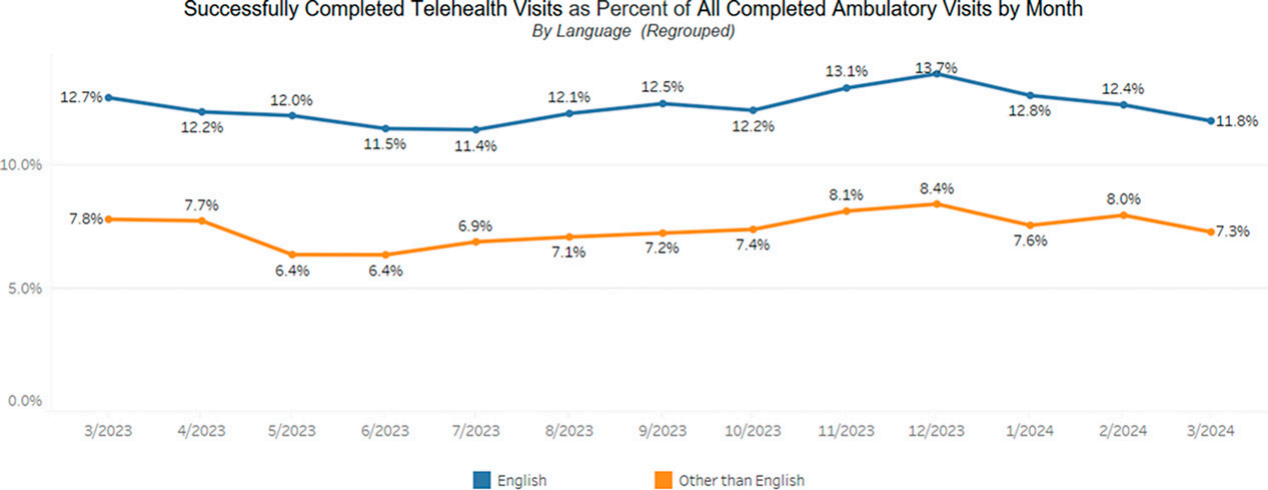
A run chart of the outcome measure (“Successfully Completed Telehealth Visits as Percent of All Completed Ambulatory Visits by Month”) stratified by patient language preference. The data are drawn from a random combination of clinics.

For each measure, the data are presented as a stacked bar graph, showing both telephone visits and video visits. The dashboard also includes a fourth section that is a run chart of the outcome measure, allowing viewers to easily visualize changes in data patterns over time. Figures 1 and 2 illustrate example data for the dashboard. Figure 1 displays the process measure categorized by race/ethnicity, and Figure 2 shows a run chart categorized by preferred language.

Measures are stratified by patient age, insurance, race and ethnicity, language preference, and neighborhood health condition.^15^ The dashboard also allows users to specify population, timeframe, department, clinic, and provider. It includes sharing capabilities through icons for downloading and sharing screenshots for professional collaboration. The dashboard retrieves data through structured query language (SQL) code sourced from the Epic electronic health record (EHR). The dashboard displays real-time data and is widely accessible to the members of our health system via Tableau, a secure data visualization software.

## Dashboard Data Validation

Four subject matter experts who are familiar with Epic EHR documentation worked with technical experts to curate the data. Data curation involved sourcing data from the EHR and applying business rules into the SQL coding logic. For example, through discussion, the team determined what constituted a “successful” versus “failed” visit. The team defined these terms and the associated SQL coding logic through a series of collaborative sessions from January to December 2022. The team then detailed the validation process in a series of collaborative planning sessions in late December 2022. The validation objectives were to achieve parity with other similar health system reports, identify and explore potential outlier scenarios, and validate data against patient charts in the EHR. The team sampled a random selection of 200 charts for successfully completed visits and 50 charts for failed visits. The team compared data—including encounter type, patient demographics, insurance, California Healthy Places Index (CA HPI) quartile,^15^ visit completion status—between the dashboard and EHR, ensuring accurate data curation and preventing misinterpretation in the dashboard.

The comparison process identified discrepancies and allowed our team to resolve identified issues. Issues included discrepancies regarding race and ethnicity categorization, distinguishing gender from sex, incomplete CA HPI data, and missingness for preferred language. Our team investigated to reveal the source of each issue and subsequently adjusted the SQL code. Ultimately, validation efforts yielded 97.2% accuracy. Regarding resources, this process required an estimated 2–5 min per encounter per person. Overall, this validation process was performed from January to July 2023.

## Iterative Improvements Based on End User Feedback

After optimizing data accuracy, we gathered feedback from stakeholders, including clinic and telehealth program managers, researchers, and communication specialists. We sought feedback to enhance its usability and relevance. Suggestions included adjusting verbiage, graph colors, and titles to improve readability and interpretation. We iteratively refined the dashboard content, layout, and labels until no further issues or suggestions for changes were identified.

## Implications

Our telehealth equity dashboard development and validation processes illustrate the potential of dashboards to enhance telehealth accessibility in health care systems. These dashboards offer institution-specific data, helping stakeholders identify areas for improvement. This insight enables health system leaders to understand existing access disparities among patient populations, a pivotal step in QI efforts for advancing health equity.^13^ This strategic dashboard usage acts as an informative tool and catalyst for targeted interventions, paving the way for impactful enhancements in telehealth accessibility and service delivery.

Teams can utilize the dashboard to systematically monitor outcome, process, and balance measures across groups. Sustained data monitoring enables iterative adjustment to interventions, optimizing project outcomes. This approach underscores a commitment to adaptive strategies and continuous improvement, helping teams align with their institution’s best practices and specifical health care improvement goals for their community.

The utilization of data for tailored interventions extends beyond telehealth, permeating multiple health care areas. Clinics can adapt interventions based on their data, priorities, and resources, to support patients in underserved communities. Identifying specific factors affecting these populations is essential for optimizing telehealth and easing the burden on medical providers. This focused, adaptable approach contributes to lasting improvements that tackle immediate challenges and promote long-term improvements.

Upon widely disseminating the telehealth equity dashboard to members across the health system, our team received feedback that the dashboard is user-friendly and innovative. The dashboard has inspired various improvement initiatives. For example, one project aims to address language-based telehealth access inequities. Project leads are partnering with community organizations to conduct focus groups with individuals who have Spanish, Russian, or Cantonese language preference. Lessons learned from these focus groups will inform the key driver diagram for the QI project. The team will then conduct plan-do-study-act cycles using the dashboard’s real-time data to guide the tests of change.

In summary, the development of the telehealth equity dashboard underscores the significance of data transparency in driving continuous enhancements aimed at promoting digital health equity. We aim to carry forward the valuable lessons we’ve learned, continuing to strive to transform our health system into the most accessible and efficient resource it can be.
